# Electrolyte disorders induced by six multikinase inhibitors therapy for renal cell carcinoma: a large-scale pharmacovigilance analysis

**DOI:** 10.1038/s41598-024-56335-4

**Published:** 2024-03-07

**Authors:** Xianhua She, Donghong Yin, Qian Guo, Yang Tang, Shuyun Wang, Xuyan Wang

**Affiliations:** 1 Department of Pharmacy, Second Hospital of Shanxi Medical University, Taiyuan, Shanxi People’s Republic of China; 2 Central Laboratory, Shanxi Hospital of Integrated Traditional Chinese and Western Medicine, Taiyuan, Shanxi People’s Republic of China; 3 Key Laboratory of Research and Development of Traditional Chinese Medicine Preparations, Taiyuan, Shanxi People’s Republic of China; 4 Department of Pharmacy, School of Pharmacy, Shanxi Medical University, Taiyuan, Shanxi People’s Republic of China

**Keywords:** Multi-kinase inhibitors, Electrolyte disorders, FAERS, Real-world data, Pharmacovigilance, Cancer, Health care, Oncology

## Abstract

To provide evidence for optimization of multi-kinase inhibitors (MKIs) use in the clinic, we use the public database to describe and evaluate electrolyte disorders (EDs) related to various MKIs treated for renal cell carcinoma. We analyzed spontaneous reports submitted to the Food and Drug Administration Adverse Events Reporting System (FAERS) in an observational and retrospective manner. Selecting electrolyte disorders' adverse events to multikinase inhibitors (axitinib, cabozantinib, lenvatinib, pazopanib, sunitinib, and sorafenib). We used Reporting Odds Ratio (ROR), Proportional Reporting Ratio (PRR), Bayesian Confidence Propagation Neural Network (BCPNN), and multi-item gamma Poisson shrinker (MGPS) algorithms to analyze suspected adverse reactions of electrolyte disorders induced by MKIs (which were treated for renal cell carcinoma) between January 2004 and December 2022. As of December 2022, 2772 MKIs (which were treated for renal cell carcinoma) ICSRs were related to electrolyte disorders AEs. In general, there were more AEs cases in males, except lenvatinib and 71.8% of the cases were submitted from North America. ICSRs in this study, the age group most frequently affected by electrolyte disorders AEs was individuals aged 45–64 years for axitinib, cabozantinib, pazopanib, and sunitinib, whereas electrolyte disorders AEs were more common in older patients (65–74 years) for sorafenib and lenvatinib. For all EDs documented in ICSRs (excluding missing data), the most common adverse outcome was hospitalization(1429/2674, 53.4%), and the most serious outcome was death/life-threat(281/2674, 10.5%). The prevalence of mortality was highest for sunitinib-related EDs (145/616, 23.5%), excluding missing data (n = 68), followed by cabozantinib-related EDs (20/237, 8.4%), excluding missing data (n = 1). The distribution of time-to-onset of Each drug-related ICSRs was not all the same, and the difference was statistically significant (P = 0.001). With the criteria of ROR, the six MKIs were all significantly associated with electrolyte disorders AEs, the strongest association was the association between cabozantinib and hypermagnesaemia. MKIs have been reported to have significant electrolyte disorders AEs. Patients and physicians need to recognize and monitor these potentially fatal adverse events.

## Introduction

Renal cell carcinoma (RCC) accounts for about 3% of all cancer types. The highest prevalence of RCC occurs in Western countries, and an annual increase of about 2% has been observed over the past two decades^1^. In 2023, in the USA, RCC is expected to be the sixth and ninth most frequently diagnosed malignancy in men and women, respectively^1^.

In recent years, several tyrosine kinase inhibitors (TKIs) have been approved for use for cancer therapy^2^, especially multi-kinase inhibitors (MKIs). According to National Comprehensive Cancer Network Clinical Practice Guidelines in Oncology for Kidney Cancer set by the National Comprehensive Cancer Network (Version 4.2023) on 18 January 2023, axitinib, cabozantinib, sunitinib, lenvatinib, pazopanib, and sorafenib can be used to treat for RCC. Their efficacy, however, varies. One of their major problems is a lack of specificity, which results in a high prevalence of drug-related toxicity and therefore a reduction, withdrawal, or discontinuation of therapy.

Apart from efficacy issues, the main concern of MKIs is adverse events (AEs). Studies have indicated that the prevalence of AEs caused by MKIs ranges from 87.2 to 100%. MKIs use can cause proteinuria, diarrhea, hypertension, and palmar-plantar erythrodysesthesia^3^. Other significant side effects, including hyponatremia and other electrolyte abnormalities, have not been well characterized in patients treated with MKIs. AEs are essential for the use and success of MKI treatment. Stopping or reducing the MKI dose is detrimental to the long-term efficacy of therapy^4^. Furthermore, several studies^5–8^ stress that the timely management of AEs is key to the prognosis of patients^9^.

The US Food and Drug Administration (FDA) Adverse Event Reporting System (FAERS) is a database. The FAERS is an important tool for post-marketing safety surveillance in the USA because it gathers information regarding all AEs for the FDA, and can be used to assessment a potential safety of drugs^10^.

Employing data collected from the FAERS, we aimed to: (i) describe AEs in a more detailed manner; (ii) evaluate the reporting frequency of some toxicities through the analysis of electrolyte disorders (EDs) related to individual case safety reports (ICSRs) during therapy of MKIs against RCC (Table 1). The results of our study may be useful for the clinical optimization of RCC treatment.

**Table 1:** Summary of multikinase inhibitors (treated for renal cell carcinoma).

	First time of approval	Targeted tyrosine kinases	FDA or EMA-approved indications
Sorafenib	12/01/2005	VEGFR-2 and -3, FLT-3, PDGFR β, c-KIT, RET, and RAF	HCC, RCC, DTC
Sunitinib	01/26/2006	VEGFR- 1, -2, -3, PDGFR, c-KIT, FLT-3	GIST, MRCC, pNET
Pazopanib	10/19/2009	FGFR, ITK, c-KIT, PDGFR, VEGFR- 1, -2, -3, LCK, and FLT-3	RCC, STS
Axitinib	01/27/2012	VEGFR- 1, -2, -3	RCC
Cabozantinib	11/29/2012	VEGFR-2, RET, MET	RCC, HCC, MTC
Lenvatinib	02/13/2015	VEGFR-1, -2, -3, FGFR-1, -2, -3, -4, PDGFR α, RET, and c-KIT	HCC, DTC, RCC, EC

*FDA* the US Food and Drug Administration, *EMA* the European Union European Medical Agency, *GIST* gastrointestinal stromal tumour, *RCC* renal cell carcinoma, *DTC* differentiated thyroid carcinoma, *HCC* hepatocellular carcinoma, *MRCC* metastatic renal cell carcinoma, *pNET* pancreatic neuroendocrine tumours, *MTC* medullary thyroid cancer, *EC* endometrial carcinoma, *VEGFR* vascular endothelial growth factor receptor, *PDGFR* platelet-derived growth factor receptor, *c-KIT* the stem cell factor receptor, *MET* the hepatocyte growth factor receptor, *FLT-3* FMS-like tyrosine kinase 3, *EGFR* the epidermal growth factor receptor, *RET* rearranged during transfection, *FGFR* fibroblast growth factor receptor1, *RAF* rapidly accelerated fibrosarcoma, *STS* advanced soft tissue sarcoma.

## Materials and methods

### Study design and data sources

This observational and retrospective pharmacovigilance study was a disproportionality analysis according to the individual case safety reports (ICSRs) taken from the FAERS (from quarter-1 in 2004 to quarter-4 in 2022). The FAERS dataset we used comprised seven data tables containing: demographic information (e.g., sex, age, bodyweight), source and type of the ICSR; country in which the ICSR was reported, dates of starting and ending (if available) of drug use. AEs and their outcomes; indications of use. We investigated the potential association of EDs with MKI use for RCC. Ethical approval was not required because this study was conducted using de-identified data.

### Drugs and adverse event identifcation

In the FAERS, AEs were coded using the preferred terms (PTs) and System Organ Classes (SOCs) in the Medical Dictionary for Regulatory Activities (MedDRA). The Standardised MedDRA Query (SMQ) is a comprehensive, validated, pre-defined set of Preferred Terms to assist regulators and drug companies in dealing with pharmaceutical safety issues. A PT is defined as the distinct descriptor for a single medical concept. Twenty PTs of EDs (“Blood calcium abnormal”, “Blood calcium decreased”, “Blood calcium increased”, “Blood magnesium abnormal”, “Blood magnesium decreased”, “Blood magnesium increased”, “Blood phosphorus decreased”, “Blood potassium abnormal”, “Blood potassium decreased”, “Blood potassium increased”, “Blood sodium abnormal”, “Blood sodium decreased”, “Hypercalcaemia”, “Hyperkalaemia”, “Hypermagnesaemia”, “Hypocalcaemia”, “Hypokalaemia”, “Hypomagnesaemia”, “Hyponatraemia”, “Hypophosphataemia”) were identified in the database.

The drugs of interest were axitinib, cabozantinib, sunitinib, lenvatinib, pazopanib, and sorafenib, as recommended in the Clinical Practice Guidelines in Oncology for Kidney Cancer set by the National Comprehensive Cancer Network (version 4.2023).

### Descriptive analysis

For this study, the following data were retrieved from FAERS—the patient’s sex and age at the time of adverse event, the type of reporter (health professional or not), reporting year and country, the event, as well as its occurrence date, seriousness criteria and the outcome of the event. The following variables concerning MKIs were also extracted-drug name, drug role in event occurrence (suspect drug, concomitant or interacting), and prescription dates when available. A major problem in spontaneous reporting data is the presence of duplicates (i.e., the same report submitted by different sources) and multiple reports (i.e., a follow-up of the same case with additional and updated information). In the present study, a two-step procedure of deduplication was applied. Firstly, only the last version of cases for which a follow-up was available was used. Secondly, cases with the same event, event date, age, gender, and country of origin were considered duplicated^11^. The reports in which the reported date of the start of a drug was after the date of the AEs were considered aberrant and excluded from the analysis.

### Statistical analyses and signal detection

A descriptive analysis of all the ICSRs was conducted to assess the demographic characteristics and variables related to the drugs under study. The analyses were performed considering the demographic data of ICSRs (gender and age), reporter country, indication, time to report, and outcome of AEs. The outcome of the AEs was classified as” Death”, “Death/ Disability/ Other “, “Disability”, “Disability/ Congenital Anomaly/ Other”, et, al. In the case of two or more AEs with different results reported in a single ICSR, the result with the lowest resolution level was chosen for classification. The calculation of the time-to-onset of EAD events was carried out according to the following formula—(Time-to-onset = Event onset date − Therapy start date).

Using a reporting odds ratio (ROR) method^12,13^ and the proportional reporting ratio (PRR), bayesian confidence propagation neural network (BCPNN) method^14^, and the multi-item gamma Poisson shrinker (MGPS) method to evaluate the potential association between the target drug and AEs. ROR and its 95% confidence interval, PRR, BCPNN, and MGPS are calculated based on the four-grid table of proportional imbalance measurement (Table 2). The equations and criteria^15^ for the above four algorithms are shown in Table 3. At least one of the four algorithms meets the standard and should be regarded as a risk signal. All the calculations were performed with Microsoft Excel 2021(Microsoft Corporation, Redmond, WA, USA).

**Table 2:** Two-by-two contingency table for analysis.

Drugs	Electrolyte disorders adverse reactions cases	All other adverse event cases	Total
Multikinase inhibitors (treated for renal cell carcinoma)	A	b	a + b
All other drugs	C	d	c + d
Total	a + c	b + d	a + b + c + d

**Table 3:** Summary of major algorithms used for signal detection.

Algorithms	Indicator	Equation	Criteria
ROR	ROR	ROR = ad/c/b	ROR05 > 1, N ≥ 2
		95CI = e^ln(ROR)±1.96(1/a+1/b+1/c+1/d)^0.5^	
PRR	PRR	PRR = a/(a + b)/c/(c + d)	PRR ≥ 2
	χ^2^	χ^2^ = [(ad-bc)^2^(a + b + c + d)]/[(a + b)(c + d)(a + c)(b + d)]	χ^2^ ≥ 4, N ≥ 3
BCPNN	IC	IC = log_2_[a(a + b + c + d)]/[(a + c)(a + b)]	IC025 > 0
		95CI = e^ln(IC)±1.96(1/a+1/b+1/c+1/d)^0.5^	
MGPS	EBGM	EBGM = a(a + b + c + d)/(a + c)/(a + b)	EBGM05 > 2, N ≥ 0
		95CI = e^ln(EBGM)±1.96(1/a+1/b+1/c+1/d)^0.5^	

*N* number of adverse event reports, *CI* confidence interval, *ROR* reporting odds ratio, *ROR05* the lower limit of the 95 two-sided CI of the ROR, *N* the number of co-occurrences, *PRR* proportional reporting ratio, *χ*^*2*^ chi-squared, *BCPNN* Bayesian confidence propagation neural network, *IC* information component, *IC025* the lower limit of the 95 two-sided CI of the IC, *MGPS* multi-item gamma Poisson shrinker, *EBGM* empirical Bayesian geometric mean, *EBGM05* the lower 95 two-sided CI of EBGM.

## Results

### Demographic characteristics

**Figure 1:**
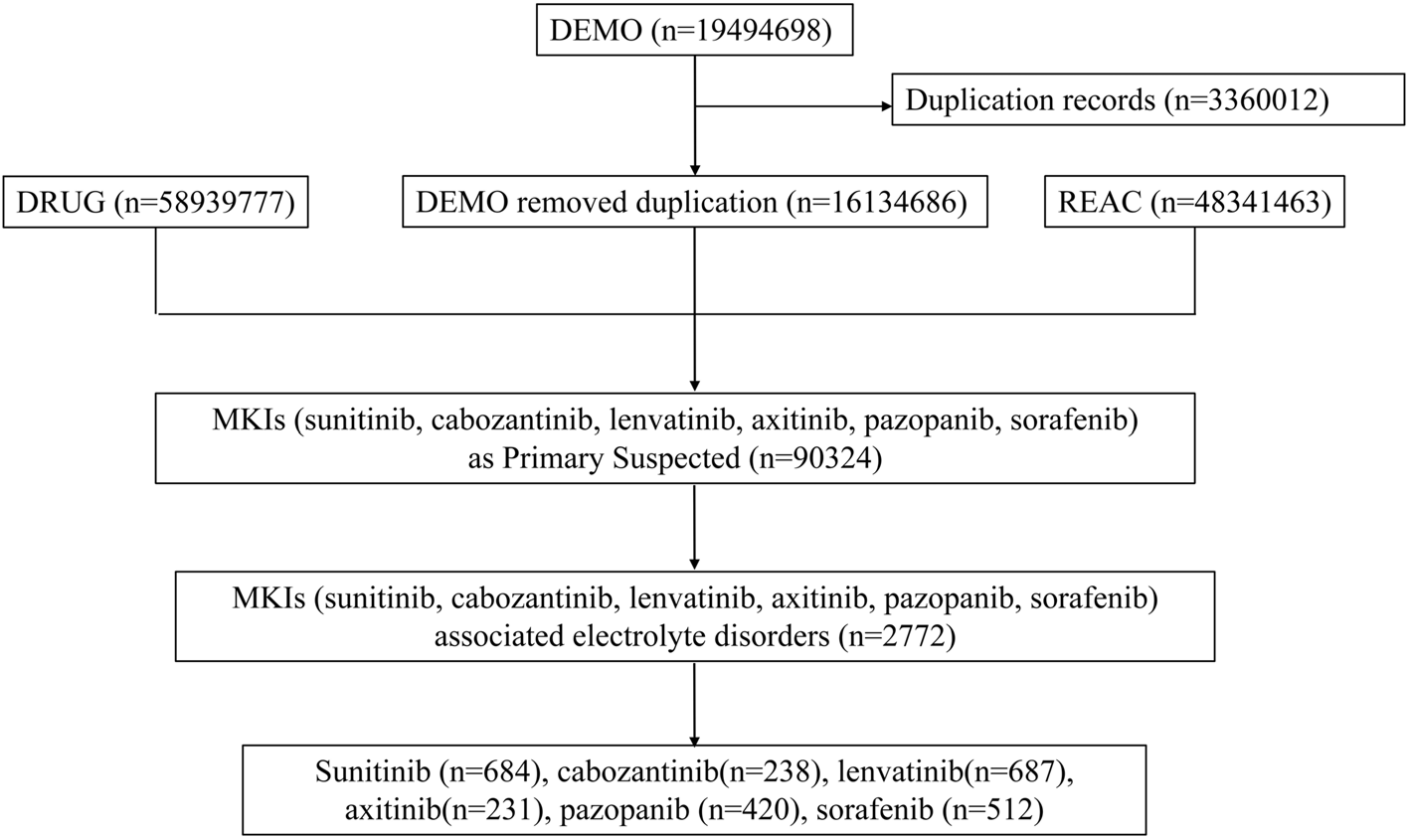
Flow chart of selection.

**Figure 2:**
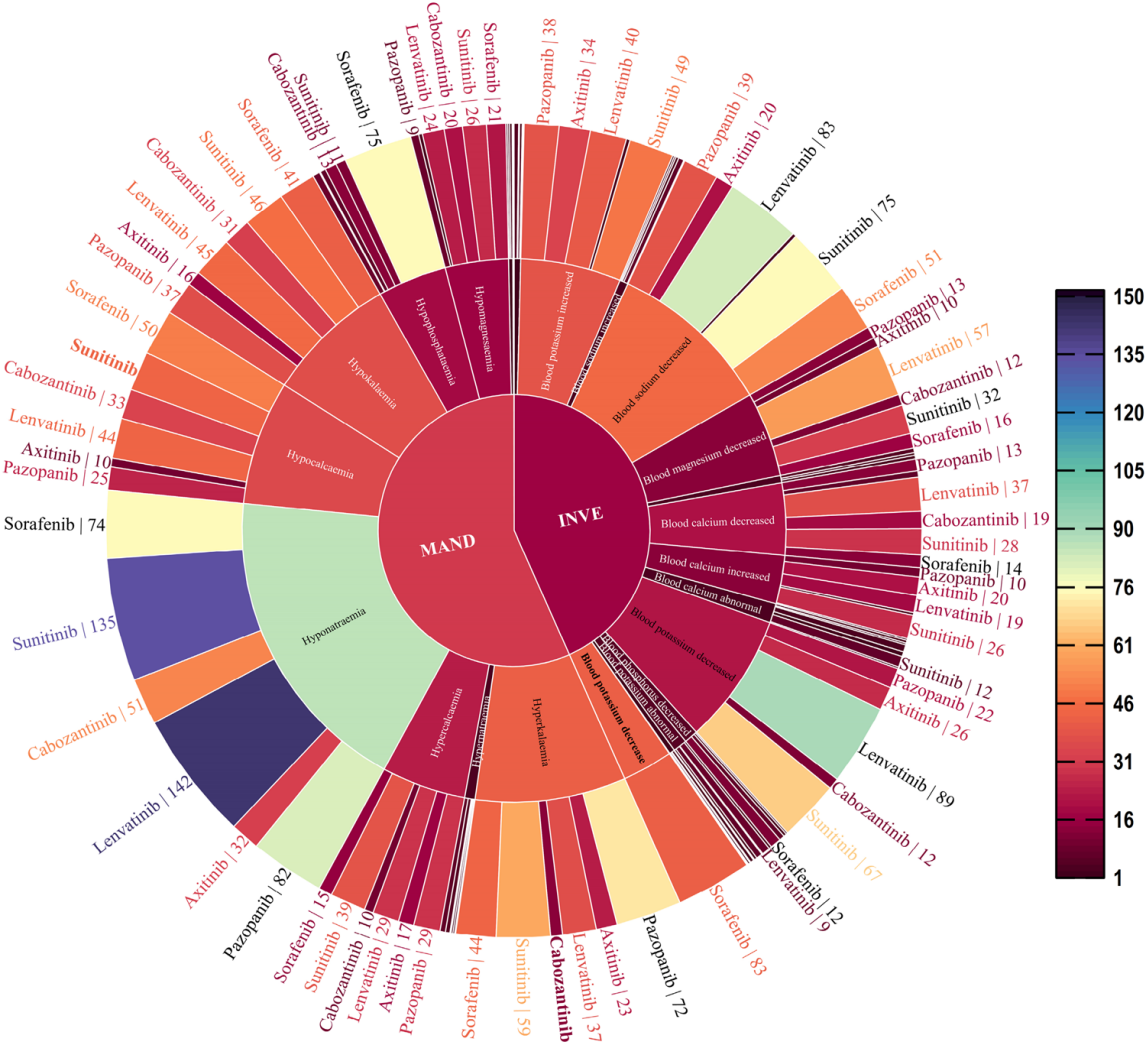
Sunburst plot of target drugs grouped by system organ classes (SOCs) classification. *MAND* metabolism and nutrition disorders, *INVE* investigations.

From January 2004 to December 2022, the FAERS database contained 19,494,698 ICSRs, of these, 16,134,686 ICSRs were retained after duplicated records had been excluded. A total of 90,324 ICSRs (sunitinib 21,840, cabozantinib 4324, lenvatinib 13,802, axitinib 12,697, pazopanib 23,265, sorafenib 14,396) suspected to be related to MKIs had been reported. Also, 2772 ICSRs were related to AEs involving EDs. Specifically, 687 (24.78%) ICSRs were linked to lenvatinib, 684 (24.68%) to sunitinib, 512 (18.47%) to sorafenib, 420 (15.15%) to pazopanib, 238 (8.59%) to cabozantinib, and 231 (8.33%) to axitinib (Fig. 1). As shown in Fig. 2, there were two SOCs (investigations, metabolism and nutrition disorders) and 26 PTs, of which 15 and 11 were associated with Investigations and Metabolism and nutrition disorders, respectivel. Fifteen PTs, blood calcium decreased, blood calcium increased, blood calcium abnormal, blood potassium increased, blood magnesium decreased, blood potassium decreased, hyperkalaemia, hypercalcaemia, blood sodium decreased, hypernatraemia, hyponatraemia, hypocalcaemia, hypokalaemia, hypophosphataemia, hypomagnesaemia, were reported for all six drugs. Four PTs, blood sodium abnormal, blood sodium increased, blood potassium abnormal, and blood magnesium increased, were reported for five drugs. Blood magnesium increased was reported for axitinib (n = 1), lenvatinib (n = 4), cabozantinib (n = 1), sunitinib (n = 2), sorafenib (n = 1); blood potassium abnormal was reported for pazopanib (n = 3), lenvatinib (n = 9), cabozantinib (n = 1), sunitinib (n = 7), sorafenib (n = 3); blood sodium abnormal and blood sodium increased was reported for pazopanib (n = 5), axitinib (n = 1), lenvatinib (n = 3), sunitinib (n = 1), sorafenib (n = 1). A reduction in the phosphorus level in blood was reported for four drugs, pazopanib (n = 2), lenvatinib (n = 3), sunitinib (n = 6), sorafenib (n = 12). Three PTs, (abnormal magnesium level in blood, increase in the phosphorus level in blood, hypermagnesemia) reported for three drugs, blood magnesium abnormal reported for lenvatinib (n = 4), cabozantinib (n = 1), sunitinib (n = 3); Blood phosphorus increased reported for pazopanib (n = 5), axitinib (n = 3), sunitinib (n = 4); Hypermagnesaemia reported for pazopanib (n = 2), lenvatinib (n = 2), cabozantinib (n = 3). One PT, blood phosphorus abnormal reported for lenvatinib (n = 1), sunitinib (n = 1). Hyperphosphataemia was reported for lenvatinib (n = 1). Hyponatraemic syndrome reported for sunitinib (n = 1).

Data on EDs after use of sorafenib or sunitinib were documented in ICSRs from 2006, and the highest number of ICSRs was in 2010 and 2015, respectively. For pazopanib, data on EDs were collected from 2010, and the highest number of ICSRs was in 2019. For axitinib, data on EDs were collected from 2012, and the highest number of ICSRs was in 2022. For cabozantinib, data on EDs were collected from 2013, and the highest number of ICSRs was in 2020. For lenvatinib, data on EDs were collected from 2015, and the highest number of ICSRs was in 2020 and 2022. The total number of ICSRs based on EDs was highest in 2019.

In general, more males suffered AEs (with the exception of lenvatinib use) and 71.8% of cases were from North America. For individuals taking axitinib, cabozantinib, pazopanib, and sunitinib, those aged 45–64 years were affected most frequently by EDs. But for sorafenib and lenvatinib, those aged 65–74 years were most. Sunitinib is most commonly used for RCC; lenvatinib is most commonly used for therapying, thyroid cancer; pazopanib, sorafenib, and axitinib are most commonly used for liver cancer. Overall, MKIs are most commonly used for liver cancer, followed by RCC. For all EDs documented in ICSRs (excluding missing data), the most common adverse outcome was hospitalization (1429/2674, 53.4%), and the most serious outcome was death/life-threat (281/2674, 10.5%). The prevalence of mortality was highest for sunitinib-related EDs (145/616, 23.5%), excluding missing data (n = 68), followed by cabozantinib-related EDs (20/237, 8.4%), excluding missing data (n = 1). As shown in Table 4.

**Table 4:** Demographic and clinical data of electrolyte disorders with interested multikinase inhibitors.

	Axitinib ( N = 231 )		Cabozantinib ( N = 238 )		Lenvatinib ( N = 687 )		Pazopanib (N = 420)		Sunitinib ( N = 684 )		Sorafenib ( N = 512)		Total (N = 2772)	
	N	%	N	%	N	%	N	%	N	%	N	%	N	%
Sex														
Females	96	41.6	93	39.1	422	61.4	149	35.5	259	37.9	162	31.6	1181	42.6
Males	124	53.7	117	49.2	258	37.6	245	58.3	394	57.6	343	67.0	1481	53.4
Missing	11	4.76	28	11.8	7	1.0	26	6.2	31	4.5	7	1.4	110	4.0
Age distribution (years)														
<18	0	0.0	2	0.8	4	0.6	5	1.2	0	0.0	4	0.8	15	0.5
18–44	5	2.2	17	7.1	7	1.0	11	2.6	34	5.0	12	2.3	86	3.1
45–64	93	40.3	70	29.4	238	34.6	121	28.8	251	36.7	167	32.6	940	33.9
65–74	74	32.0	55	23.1	265	38.6	110	26.2	213	31.1	174	34.0	891	32.1
75–84	34	14.7	35	14.7	99	14.4	45	10.7	113	16.5	118	23.1	444	16.0
≥ 85	4	1.7	5	2.1	12	1.8	11	2.6	9	1.3	8	1.6	49	1.8
Other	21	9.1	54	22.7	62	9.0	117	27.9	64	9.4	29	5.7	347	12.6
Reporter country														
North America	143	61.9	148	62.2	433	63.0	170	40.5	368	53.8	205	40.0	1467	52.9
South America	6	2.6	2	0.8	3	0.4	10	2.4	46	6.7	22	4.3	89	3.2
Europe	20	8.7	80	33.6	81	11.8	125	29.8	123	18.0	74	14.5	503	18.1
Asia	62	26.8	6	2.5	158	23.0	83	19.8	135	19.7	207	40.4	651	23.5
Other	0	0.0	1	0.4	7	1.0	6	1.4	9	1.3	4	0.8	27	1.0
Missing	0	0.0	1	0.4	5	0.7	26	6.2	3	0.4	0	0.0	35	1.3
Outcome														
Death/life-threat	17	7.4	20	8.4	39	5.7	25	6.0	145	21.2	35	6.8	281	10.1
Disability	0	0.0	3	1.3	0	0.0	1	0.2	4	0.6	2	0.4	10	0.4
Hospitalization	75	32.5	125	52.5	549	79.9	143	34.1	364	53.2	173	33.8	1429	51.6
Other serious	130	56.3	89	37.4	99	14.4	241	57.4	103	15.1	292	57.0	954	34.4
Missing	9	3.9	1	0.4	0	0.0	10	2.4	68	9.9	10	2.0	98	3.5
Indication														
Renal cancer	149	64.5	71	29.8	90	13.1	146	34.8	83	12.1	257	50.2	796	28.7
Hepatic cancer	0	0.0	11	4.6	95	13.8	1	0.2	387	56.6	0	0.0	494	17.8
Thyroid cancer	1	0.4	67	28.2	160	23.3	7	1.7	19	2.8	1	0.2	255	9.2
EC/EOC	0	0.0	0	0.0	2	0.3	1	0.2	2	0.3	127	24.8	132	4.8
CRC	0	0.0	13	5.5	130	18.9	4	1.0	0	0.0	0	0.0	147	5.3
Other	81	35.1	76	31.9	210	30.6	261	62.1	193	28.2	127	24.8	948	34.2
Reported time-to-onset (days)														
0–30	18	7.8	36	15.1	62	9.0	89	21.2	94	13.7	121	23.6	420	15.2
31–60	11	4.8	14	5.9	18	2.6	31	7.4	40	5.9	15	2.9	129	4.7
61–90	2	0.9	10	4.2	14	2.0	17	4.1	19	2.8	10	2.0	72	2.6
91–180	9	3.9	21	8.8	26	3.8	17	4.1	22	3.2	14	2.7	109	3.9
181–360	10	4.3	15	6.3	10	1.5	13	3.1	11	1.6	8	1.6	67	2.4
> 360	3	1.3	3	1.3	13	1.9	20	4.8	37	5.4	14	2.7	90	3.3
Other	30	13.0	51	21.4	103	15.0	67	16.0	47	6.9	145	28.3	443	16.0
Missing	148	64.1	88	37.0	441	64.2	166	39.5	414	60.5	185	36.1	1442	52.0

*EC* endometrial cancer, *EOC* epithelial ovarian cancer, *CRC* colorectal cancer.

**Figure 3:**
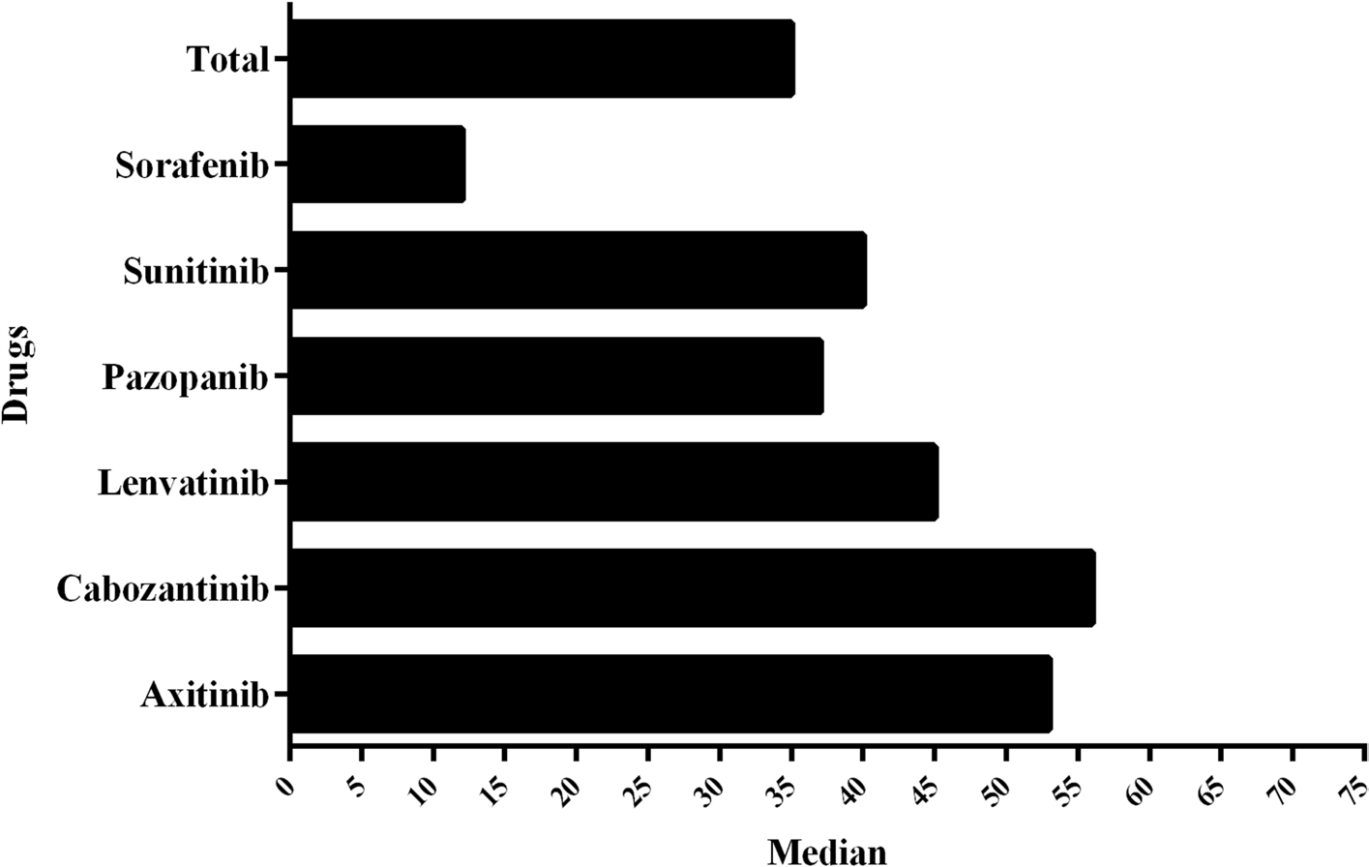
Median value of time-to-onset from MKIs used to electrolyte disorders events occurrence.

A total of 887 cases were suitable for calculating the median value of time to onset of the ICSRs. We found that the median time to onset of axitinib-related ICSRs was 53 days, cabozantinib-related ICSRs was 56 days, lenvatinib-related ICSRs was 45 days, pazopanib-related ICSRs was 37 days, sunitinib-related ICSRs was 40 days, sorafenib-related ICSRs was 12 days, and the overall median value of onset time was 35 days, as shown in Fig. 3.

**Figure 4:**
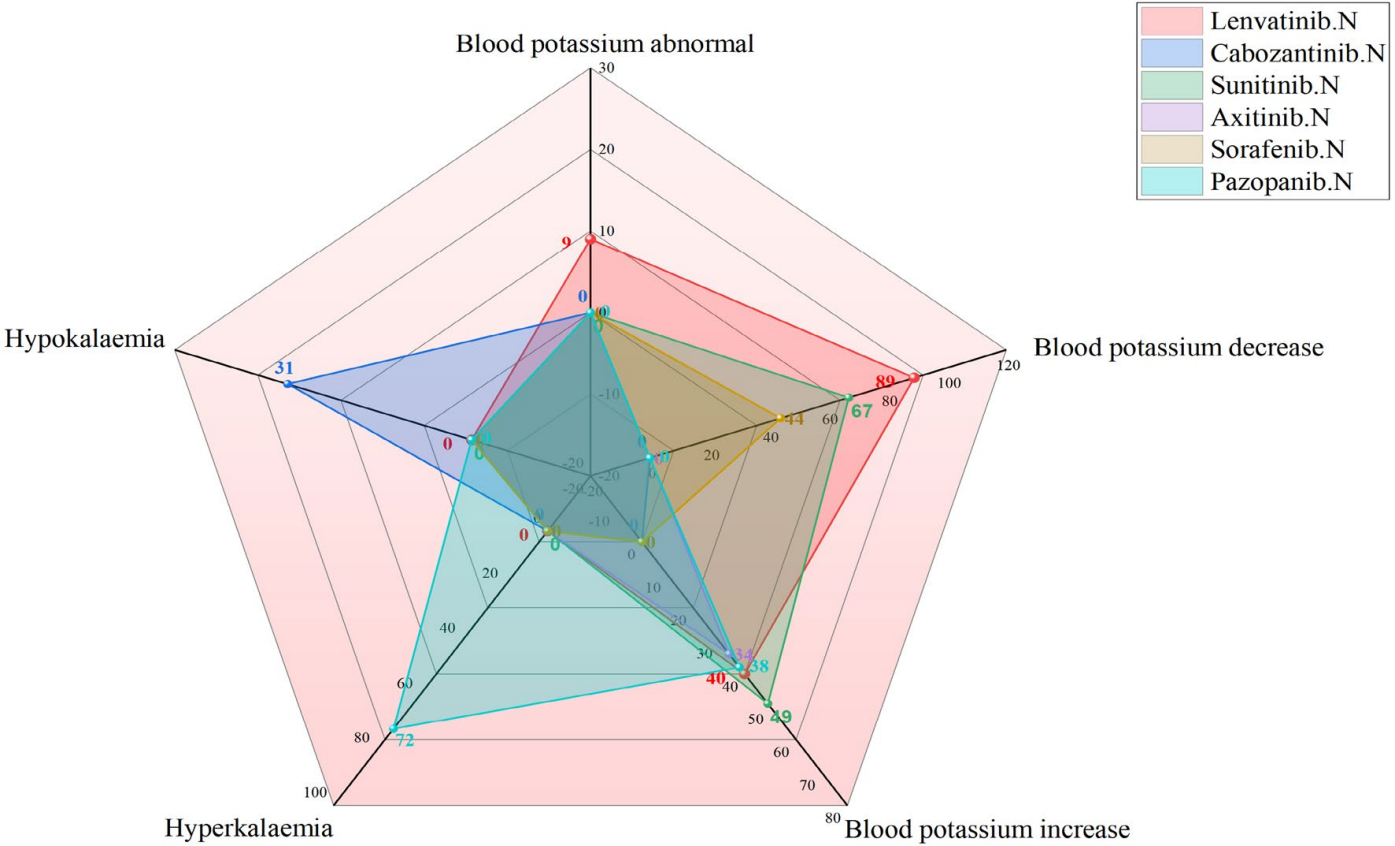
Case number and PTs distribution of electrolyte disorders (potassium) to curated candidate drugs.

**Figure 5:**
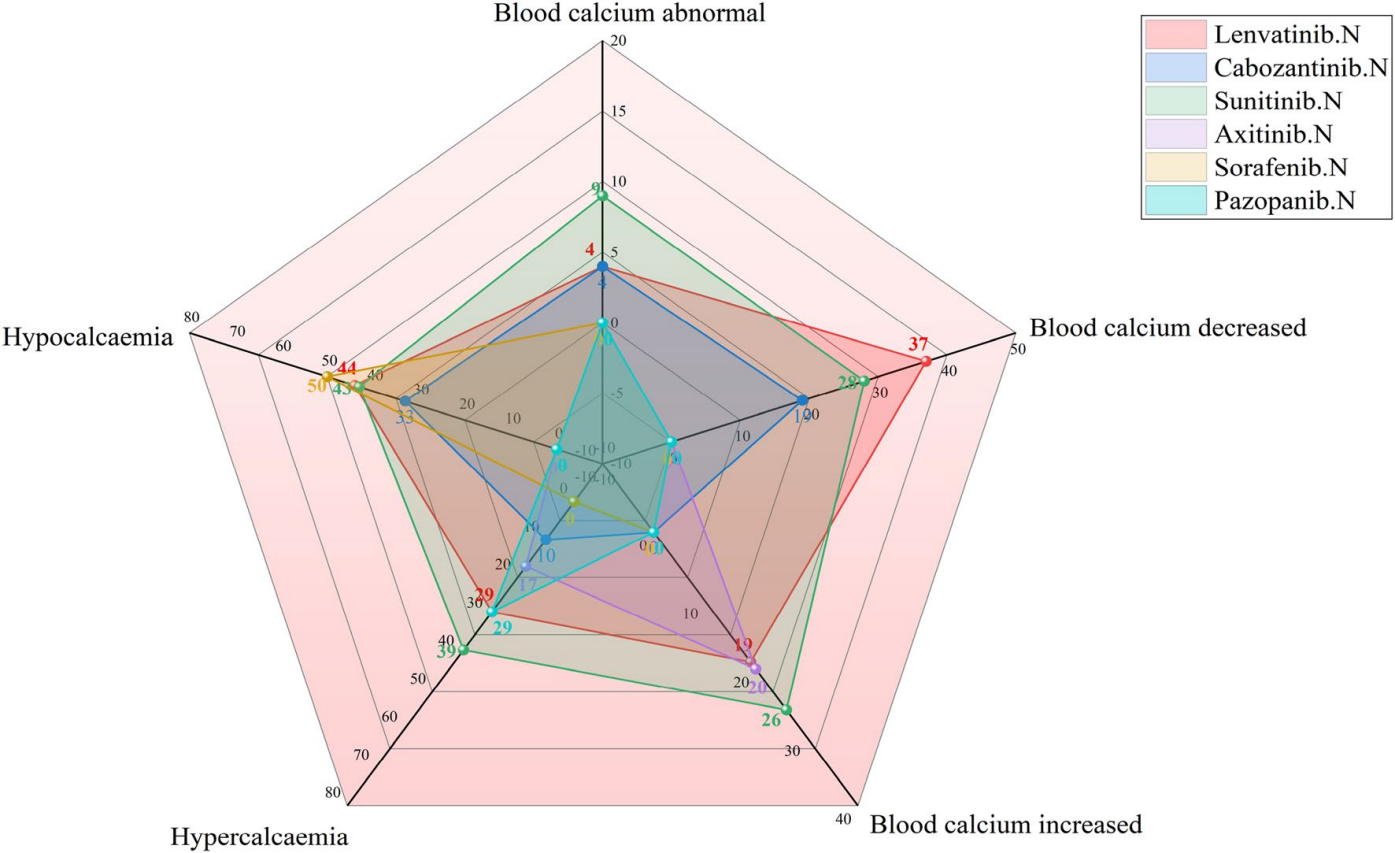
Case number and PTs distribution of electrolyte disorders (calcium) to curated candidate drugs.

**Figure 6:**
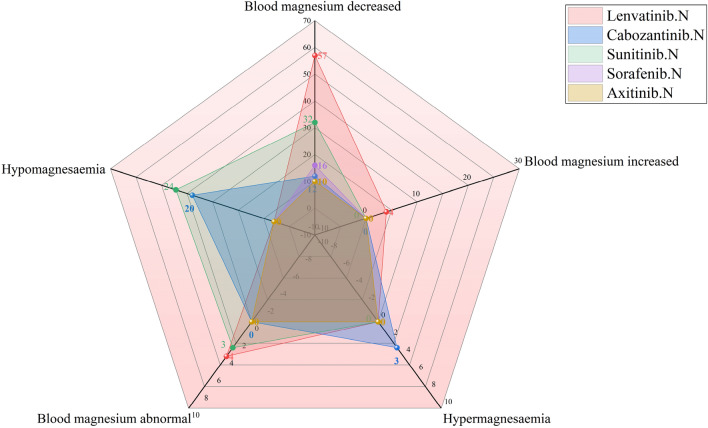
Case number and PTs distribution of electrolyte disorders (magnesium) to curated candidate drugs.

**Figure 7:**
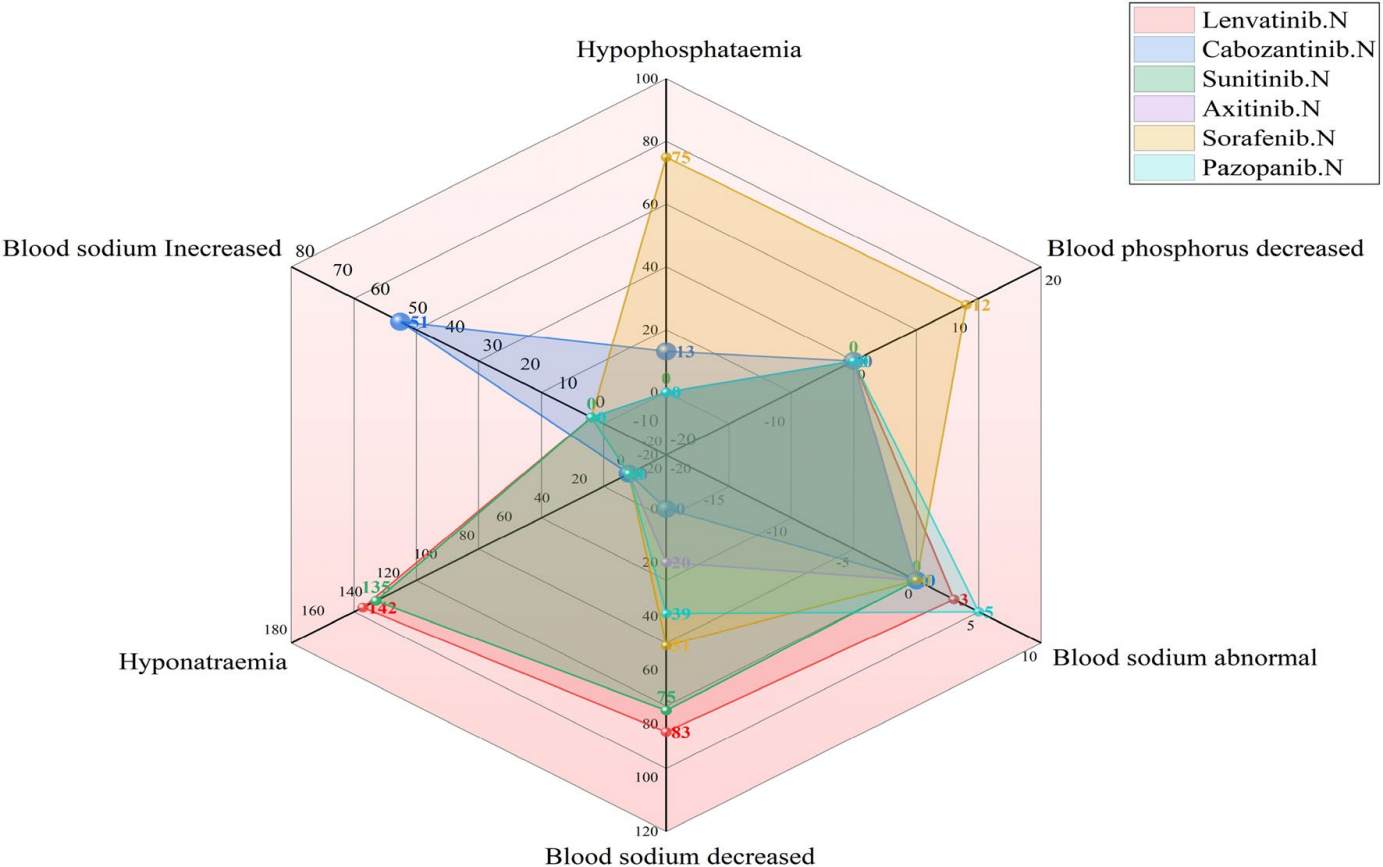
Case number and PTs distribution of electrolyte disorders (sodium and phosphorus) to curated candidate drugs.

As shown in Fig. 4, hyponatremia or a reduction in the sodium level in blood was highest after use of lenvatinib (documented in 142 and 83 ICDRs, respectively), followed by sunitinib use (135 and 75, respectively). Hypophosphataemia or blood phosphorus decreased, was highest after sorafenib use (documented in 75 and 12 ICSRs, respectively). As shown in Fig. 5, among the AEs of the electrolyte potassium associated with MKIs, lenvatinib-related blood potassium decrease ICSRs was the most (n = 89), but cabozantinib-related hypokalaemia ICSRs were the most (n = 31); sunitinib-related blood potassium increase ICSRs was the most (n = 49), but pazopanib-related hyperkalaemia ICSRs was the most (n = 72). As shown in Fig. 6, among the AEs of the electrolyte calcium associated with MKIs, lenvatinib-related blood calcium decreased ICSRs was the most (n = 37), but sorafenib-related hypocalcaemia ICSRs were the most (n = 50); whether hypercalcaemia or blood calcium increased, sunitinib-related ICSRs was the most (39 and 26, respectively). As shown in Fig. 7, among the AEs of the electrolyte magnesium associated with MKIs, lenvatinib-related blood magnesium decreased ICSRs were the most (n = 57), but sunitinib-related hypomagnesaemia ICSRs were the most (n = 24); lenvatinib-related blood magnesium increased ICSRs was the most (n = 4), only cabozantinib-related hypermagnesaemia ICSRs was reported (n = 3). As shown in Table 4.

### Signal values of electrolyte disorders AEs associated with MKIs

**Figure 8:**
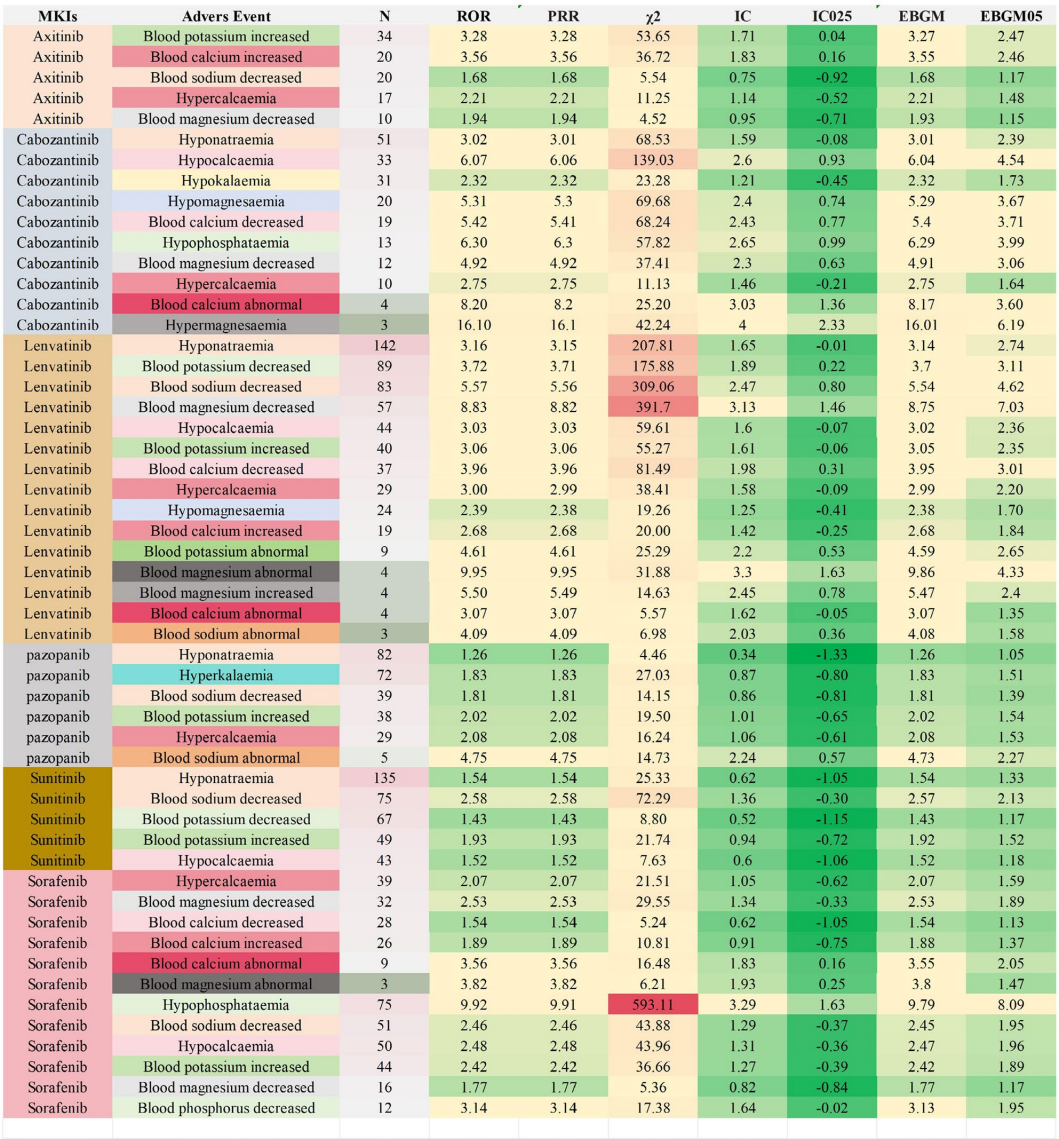
Electrolyte disorders with disproportionality signals for the six multikinase inhibitors.

As shown in Fig. 8, with the criteria of ROR, the six MKIs were all have a significant association with ED AEs, the strongest was cabozantinib and hypermagnesaemia (ROR = 16.10), followed by lenvatinib and blood magnesium abnormal (ROR = 9.95). Based on ROR, PRR, BCPNN, and MGPS methods, axitinib and blood potassium increased, and blood calcium increased; cabozantinib and hypocalcaemia, hypomagnesaemia, blood calcium decreased, hypophosphataemia, blood magnesium decreased, blood calcium abnormal, and hypermagnesaemia; lenvatinib and blood potassium decreased, blood sodium decreased, blood magnesium decreased, blood calcium decreased, blood potassium abnormal, blood magnesium abnormal, and blood magnesium increased; pazopanib and blood sodium abnormal; sunitinib and blood calcium abnormal; sorafenib and hypophosphataemia have a significantly associated between them.

## Discussion

This is the first study investigating the relationship between MKIs and the risk of EDs using a pharmacovigilance approach. MKIs are used widely to treat RCC, and EDs are encountered commonly in patients suffering from cancer. EDs can lead to life-threatening complications^16,17^.

Spontaneous reporting systems are required for the early identification and characterization of individual AEs in real-time. Such data support awareness among oncologists regarding the need to manage those AEs promptly.

Over the years, the distribution of AEs has been influenced by the different timings of approval and clinical use of TKIs^18^. In the present study, the main indications for MKI use were liver cancer and RCC (28.72% and 17.82%, respectively). Males and those aged 45–74 years were the main groups affected by EDs in our study. A causal relationship between EDs and MKI use has not been shown. Studies have demonstrated a marked association between hyponatremia and mortality in patients with non-Hodgkin’s lymphoma, RCC, gastric cancer, and small-cell lung cancer^14^.

This study found that the onset of electrolyte disturbances varies greatly, ranging from a few days to several years after starting MKI treatment. The onset time of EDs induced by sorafenib is lower than that of other drugs. This result may be one of the reasons why the panel consensus which mentioned in the National Comprehensive Cancer Network Clinical Practice Guidelines in Oncology for Kidney Cancer (Version 4.2023—January 18, 2023) did not support the inclusion of sorafenib as a subsequent therapy option for RCC. Although there was a significant increase in overall survival (OS) and progression-free survival (PFS) in patients treated with sorafenib compared to placebo.

EDs are common in cancer patients and may be secondary to the cancer^19^, specific pharmacodynamic mechanisms, or acute renal impairment, albuminuria, and hypertension. For example, some antineoplastic agents may interfere with the tubular effects of electrolytes and urinary excretion by interfering with antidiuretic hormones. Hyponatremia is the most frequently observed EDs in cancer patients. The major cause of hyponatremia is volume depletion, which is most commonly associated with bleeding, diarrhea, vomiting, ascites, pleural effusion, peritonitis, or ileus^20^. Syndrome of inappropriate antidiuretic hormone (SIADH) is considered to be another frequent cause of hyponatremia in cancer patients^21^. Dose-dependent use of axitinib has been associated with hyponatremia. The use of sorafenib, sunitinib^22^, pazopanib^23^ has also been associated with a high prevalence of hyponatremia. The major underlying mechanism is SIADH induction^24–26^. In the present study, ICSRs for hyponatremia were documented for all six medications.

The release of phosphorus from damaged cells and presence of extracellular phosphorus causes hyperphosphatemia, as noted in tumorlysis syndrome, rhabdomyolysis, lactic acidosis, ketoacidosis, and respiratory alkalosis^21^. Use of axitinib, sunitinib, and sorafenib has been associated with hypocalcemia in patients with advanced cancer^27,28^. Sunitinib, by inhibiting bone turnover, interferes with phosphate homeostasis. Sorafenib can influence bone turnover through the inhibition of platelet-derived growth factor receptors and the induction of acquired Fanconi syndrome^25^. Hypophosphatemia can be worsened by concomitant conditions, and induce diarrhea upon consequent malabsorption of vitamin D^19,29^. In the present study, ICSRs for hyponatremia were documented for all six MKIs.

Most cancer patients undergo targeted therapy and chemotherapy (which can cause nausea and vomiting). Excessive vomiting (especially for long periods) results in hypovolemia and hypochloremic metabolic alkalosis due to the loss of chloride ions and hydrogen ions, which may be related to hypokalemia and hypomagnesemia^25^. Concerning the mechanism of hypokalemia, poor nutrition, anorexia, and volume depletion may lead to insufficient intake of potassium^21^. Axitinib induces hyperkalemia via the development of tumorlysis syndrome and distal tubular dysfunction (e.g., type-4 renal tubular acidosis during hyperkalemia^30^. In the present study, ICSRs for hyperkalemia were documented for lenvatinib, cabozatinib, sunitinib, and sorafenib.

Hypocalcemia can result from malnutrition, hypoalbuminemia, sepsis, or tumor lysis syndrome^21^. The use of sorafenib^31^, axitinib^27^, or sunitinib^32^ can cause hypocalcemia. The mechanism of the hypocalcemia elicited by sorafenib might be due to endoplasmic reticulum stress coupled with calcium mobilization. However, how sunitinib and axitinib may cause hypocalcemia is not clear. In the present study, ICSRs for hypocalcemia were documented for lenvatinib, cabozantinib, sunitinib, and sorafenib.

Magnesium has a crucial role in various physiological processes but is frequently overlooked^33^. Such lack of attention to the magnesium level may be due to: an absence of magnesium testing in routine chemistry panels; the subtlety of magnesium-deficiency symptoms; the wide range of processes in which magnesium is involved, making it challenging to attribute specific symptoms to magnesium deficiency. Hypomagnesemia is observed frequently in cancer patients and has been ascribed to malnutrition, diarrhea, hypercalcemia, and therapy with antineoplastic drugs^23^.

Interestingly, some studies have found that cabozantinib is not associated with EDs^28,34^. In our study, hyperphosphatemia, hyperkalemia, hypocalcemia, hypomagnesemia, and hyponatremia were associated with the use of cabozantinib, as well as lenvatinib and pazopanib.

Studies have reported that hypertension^11^, heart failure^35^, renal/liver insufficiency^36^, chemotherapy, hematologic malignancies, or cancer with distant metastasis can aggravate the risk of death in patients with EDs. Diabetes mellitus is an independent risk factor for hyponatremia. This indicates that EDs may be prevented by early correction of renal/hepatic impairment and other biochemical markers at the time of hospital admission. The synergistic action of antitumoral drugs and EDs can lead to fatal arrhythmias. Hence, periodic monitoring of electrolytes, an optimal mode of intravenous infusion, correction of variable factors for infusion, and appropriate management of EDs during anticancer therapy should not be overlooked, especially for those who are older, have a low body mass index, underlying disease, or receiving surgery/chemotherapy. Short-term results and quality of life could be improved by taking these measures^21^.

To obtain higher-quality evidence in this area, attention should be given to EDs caused by MKIs in randomized controlled trials. Also, more data are needed to evaluate the incidence of, and risk factor associated with, ED development. Furthermore, clarification of the mechanism of these AEs at molecular and cellular levels is needed to develop more efficacious drug therapies. There is a need for evidence-based guidance to manage EDs in patients receiving MKIs.

Our study had three main limitations. Firstly, data-mining dose not compensate for the inherent shortcomings of the FAERS, such as under, incomplete, false, and inaccurate reporting, all of which may results in a bias. The absence of laboratory values and complete medical records (including comprehensive information on concomitant medications and comorbidities) may have contributed to errors in our analysis. Second, only qualitative research could be used in our study due to the intrinsic characteristics of the FAERS. It was not possible to quantify the prevalence of EDs as AEs compared with overall AEs with MKI use. Thirdly, although the pharmacovigilance database showed a strong association between targeted drugs and their adverse reactions, it was not possible to determine whether there was a biological cause-and-effect relationship between drugs and AEs in this study because of confounding factors such as cancer and its complications, which can also cause electrolyte abnormalities^25,36^. The FAERS has limitations in terms of its inclusion of genetic factors, but it does indicate certain key features of EDs in response to MKI use, such as the timing, spectrum, and clinical manifestations of EDs, which may provide useful insights for the development of additional well-designed research.

## Conclusion

Our findings support the assumption that EDs AEs are a safety concern for the six MKIs used to treat RCC. Periodic electrolytes monitoring, optimal mode of intravenous infusion, correction of variable factors for infusion, and proper management of EDs during anticancer therapy should not be overlooked. Furthermore, a large prospective population-based study is required to determine the true incidence of AEs and to fully clarify the risk factors, which would support appropriate risk management.

## Data Availability

The datasets generated during and/or analyzed during the current study are available from the corresponding author on reasonable request.
